# Aortic rupture during reoperative bariatric surgery

**DOI:** 10.5935/1678-9741.20150051

**Published:** 2015

**Authors:** Sorin Hostiuc, Constantin Dragoteanu, Victor Asavei, Ionut Negoi

**Affiliations:** 1 Carol Davila University of Medicine and Pharmacy - Floreasca Clinical Emergency Hospital, Department of Surgery, Bucharest, Romania.; 2 National Institute of Legal Medicine, Department of Forensic Pathology, Bucharest, Romania.; 3 Polytechnic University, Bucharest, Romania.

**Keywords:** Bariatric Surgery, Obesity, Morbid, Aorta/Injuries

## Abstract

Morbid obesity has become a very common problem worldwide, causing severe health-related consequences including cardiovascular or metabolic diseases, arthritis, sleep apnea, or an increased risk of cancer. Bariatric surgery was shown to be the only way to achieve sustainable weight loss and to decrease the frequency and severity of metabolic and cardiovascular comorbidities. The purpose of this article is to present a case of bariatric surgery complicated with lesion of the aorta with a lethal outcome.

## Resumo

A obesidade mórbida é cada vez mais comum em todo o mundo, causando graves consequências relacionadas com a saúde, incluindo doenças cardiovasculares ou metabólicas, artrite, apneia do sono, ou um aumento do risco de câncer. A cirurgia bariátrica mostrou ser a única maneira de conseguir a perda de peso sustentável e para diminuir a frequência e a gravidade das comorbidades metabólicas e cardiovasculares. O objetivo deste artigo é apresentar um caso de cirurgia bariátrica que provocou lesão da aorta com um desfecho letal.

**Table:** 

**Abbreviations, acronyms & symbols**	
LAGB	Laparoscopic adjustable gastric band

## INTRODUCTION

Morbid obesity has become a very common problem worldwide, causing severe health-related consequences including cardiovascular or metabolic diseases, arthritis, sleep apnea, or an increased risk of cancer^[1]^. Bariatric surgery was shown to be the only way to achieve sustainable weight loss and to decrease the frequency and severity of metabolic and cardiovascular comorbidities^[2]^. Laparoscopic adjustable gastric band (LAGB) is in many countries, especially in Europe and Australia^[3]^, the most frequently used technique^[2]^, even though others such as the Roux-en-Y gastric bypass or biliopancreatic diversion are still performed^[2]^. The mortality rate in bariatric surgery is between 0.05 and 2%, the most frequent cause of death being an anastomotic leak with subsequent infection^[4]^. Overall, between 10 and 25% of all bariatric patients need a revision surgery for failure of the primary procedure, either determined by inadequate weight loss or surgical complications^[5]^. The mortality is increased if revision surgery is needed^[6]^.

The purpose of this article is to present a potential complication of abdominal surgery re-entry for gastric band correction at the level of the aorta having in the end a lethal outcome.

## CASE REPORT

A 26 years old woman with morbid obesity was admitted for bariatric surgery (laparoscopic adjustable gastric banding). The patient was released from the hospital after three days. After about four months she was admitted again, with an initial diagnosis of superior digestive hemorrhage and status post gastric banding. Clinically, the patient was conscious, cooperative, without signs of peritoneal irritation, with an arterial pressure of 110/60 mmHg. Radiological examination revealed an anteriorly malrotated ring, intraparietal filling with a radiopaque material, and a small retro-parietal fistula in the gastric fundus. As the patient presented superior digestive hemorrhage and ring malrotation a surgical intervention was performed, whose aim was to extract the band. Laparoscopy was performed with the trocars located above the umbilicus, in the left flank, left and right hypochondriac area, and in the epigastrium.

During the surgical intervention, the rubber hose of the ring was associated a severe inflammatory reaction, incorporating the abdominal esophagus, the cardiac area of the stomach, and the celiac region. A difficult sharp dissection was started for viscerolysis; while trying to mobilize the ring that was identified red, arterial blood flowing through the nasogastric tube. The procedure was immediately converted to a xiphoid-umbilical laparotomy associated with gastrotomy step in which were evacuated large blood clots from the stomach; the source of bleeding was found to be a laceration of about two centimeters at the anterior part of the aorta, located posteriorly from the esophagus.

At the same time the anesthetic team, noticing 600 ml of fresh blood in the aspirator of the gastric content started the resuscitation protocol. After laparotomy, another 1800 ml fresh blood was aspirated from the surgical field. There was not an available cell saver in the operating room. Due to the severity of the intraoperative incident, together with the scarce reserve of the hospital blood supplies, the patient received only four units of packed red blood cells and five units of fresh frozen plasma. The laceration was sutured but the blood losses were too great and the patient died of hemorrhagic shock, unresponsive to resuscitation maneuvers.

### Autopsy findings

**Fig. 1:**
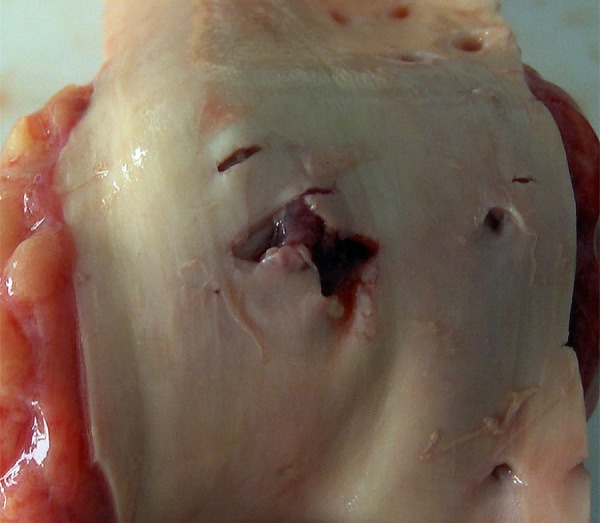
Iatrogenic aortic injury, as observed during the autopsy.

On the anterior side of the lower part of the thoracic aorta, near the diaphragm, an H laceration of about 1.2/1cm was identified, with an irregular contour and hemorrhagic infiltrate (Figure 1). On the posterior side of the aorta, corresponding to the anterior lesion, a small laceration of about 0.2 cm was identified, affecting the intima and partially the media. In the stomach, a callous ulcer near the pyloric antrum was identified on the posterior gastric wall, with rounded margins, affecting the mucosal, submucosal and muscular layer, with a diameter of about 1.5 cm. The esophagus in the subdiaphragmatic part, near the cardia, had an anfractuous laceration, affecting all the anatomical layers, with a sutured hemorrhagic infiltrate.

Histology examination confirmed the laceration, affecting all the layers of the aortic wall, with small hemorrhagic areas in the media, and abundant inflammatory reaction with lymphocytes and plasma cells, located around the vasa vasorum and in the surrounding interstitial tissue. The esophagus contained numerous, recent hemorrhagic areas mostly located between the adventitial and muscle layers, with a chronic inflammatory reaction (lymphocytes and plasma cells) in the muscle and subadventitial layers. The stomach contained an unspecific gastritis with large ulcero-necrotic areas, abundant chronic inflammatory reaction in the interstitial and perivascular areas of the submucosa, muscularis propria, and subserous layers, with immature granular tissue in the subserous layer and acute inflammatory reaction at the serous layer (non-steroidal anti-inflammatory associated gastritis). The cause of death was considered acute hemorrhage (Ia), secondary to an aortic rupture during bariatric surgery.

### Ethics

The management of ethics related issues was performed in accordance with the Romanian Law regarding the organization and functioning of the medical legal system Nº 1 from 20.01.2000, Art 2(3) and Art 15(e) and Decision No.774/2000 regarding the approval of the methodological norms for the application of the Law 1/200, Art 39(1), in full compliance with relevant international norms, including the Declaration of Helsinki. Both surgeries of the presented case were done in a tertiary surgical department, neither author being involved in the clinical management.

## DISCUSSION

Band slippage is a relatively frequent postoperative complication of LAGB, with an overall prevalence of about 5.5%^[7]^. If it is confirmed the first step is represented by band deflation through the subcutaneous port. However, in our case, the presence of superior digestive hemorrhage suggested the possibility of gastric erosion or ischemia, suggesting the need for a surgical re-intervention. During revision the surgeon must be aware that around the band is often encountered a fibrous reaction that requires careful dissection^[2]^. Moreover, aorta passes posteriorly of the stomach and esophagus, in close relation with these structures; therefore the fibrous tissue developed after bariatric surgery may have had a traction effect upon this vessel.

There is no doubt that severe chronic inflammation, with dense adhesions between the band, the abdominal esophagus, the stomach and the abdominal aorta passing through its diaphragmatic hiatus played an important role for this major incident. The characteristics of the aorta rupture suggest iatrogenic lesions during gastric dissection, most likely done by a sharp object (e.g. scizzors), that could also explain the presence of the posterior aorta lesion.

Aortic complications secondary to bariatric surgery have been rarely cited. Gaia cited a case of ruptured aortic aneurism and an aortic dissection in patients that underwent a bariatric procedure^[8]^; however they were not caused by the intervention *per se* or its complications, being most likely associated with the comorbidities of morbid obesity (atherosclerosis, aortic calcifications, and so on). This is the first reported case, to our knowledge, of an intraoperative aortic lesion secondary to a repositioning procedure for a gastric band.

## CONCLUSION

The morbid obesity surgeries, especially revision surgeries, carry a high morbidity and even mortality. Only a thorough preoperative planning, a careful operative technique, and a low threshold for conversion to open surgery may decrease the failure rate of these surgeries.

**Table:** 

**Authors’ roles & responsibilities**	
SH	Analysis and/or interpretation of data; final manuscript approval; manuscript writing or critical review of its content
CD	Analysis and/or interpretation of data; final manuscript approval; manuscript writing or critical review of its content
VA	Analysis and/or interpretation of data; final manuscript ap-proval; manuscript writing or critical review of its content
IN	Analysis and/or interpretation of data; statistical analysis; final manuscript approval; study design; manuscript writing or critical review of its content
